# Extremely low frequency electromagnetic fields promote mesenchymal stem cell migration by increasing intracellular Ca^2+^ and activating the FAK/Rho GTPases signaling pathways in vitro

**DOI:** 10.1186/s13287-018-0883-4

**Published:** 2018-05-21

**Authors:** Yingchi Zhang, Jiyuan Yan, Haoran Xu, Yong Yang, Wenkai Li, Hua Wu, Chaoxu Liu

**Affiliations:** Department of Orthopedics, Tongji Hospital, Tongji Medical College, Huazhong University of Science and Technology, Jiefang Avenue 1095, Wuhan, 430030 China

**Keywords:** Electromagnetic fields, Cell migration, Intracellular Ca^2+^, Focal adhesion kinase, Rho GTPase protein family

## Abstract

**Background:**

The ability of mesenchymal stem cells (MSCs) to migrate to the desired tissues or lesions is crucial for stem cell-based regenerative medicine and tissue engineering. Optimal therapeutics for promoting MSC migration are expected to become an effective means for tissue regeneration. Electromagnetic fields (EMF), as a noninvasive therapy, can cause a lot of biological changes in MSCs. However, whether EMF can promote MSC migration has not yet been reported.

**Methods:**

We evaluated the effects of EMF on cell migration in human bone marrow-derived MSCs. With the use of Helmholtz coils and an EMF stimulator, 7.5, 15, 30, 50, and 70 Hz/1 mT EMF was generated. Additionally, we employed the l-type calcium channel blocker verapamil and the focal adhesion kinase (FAK) inhibitor PF-573228 to investigate the role of intracellular calcium content, cell adhesion proteins, and the Rho GTPase protein family (RhoA, Rac1, and Cdc42) in EMF-mediated MSC migration. Cell adhesion proteins (FAK, talin, and vinculin) were detected by Western blot analysis. The Rho GTPase protein family activities were assessed by G-LISA, and F-actin levels, which reflect actin cytoskeletal organization, were detected using immunofluorescence.

**Results:**

All the 7.5, 15, 30, 50, and 70 Hz/1 mT EMF promoted MSC migration. EMF increased MSC migration in an intracellular calcium-dependent manner. Notably, EMF-enhanced migration was mediated by FAK activation, which was critical for the formation of focal contacts, as evidenced by increased talin and vinculin expression. Moreover, RhoA, Rac1, and Cdc42 were activated by FAK to increase cytoskeletal organization, thus promoting cell contraction.

**Conclusions:**

EMF promoted MSC migration by increasing intracellular calcium and activating the FAK/Rho GTPase signaling pathways. This study provides insights into the mechanisms of MSC migration and will enable the rational design of targeted therapies to improve MSC engraftment.

**Electronic supplementary material:**

The online version of this article (10.1186/s13287-018-0883-4) contains supplementary material, which is available to authorized users.

## Background

Mesenchymal stem cells (MSC) are present in the connective tissue that surrounds other tissues and organs, and exhibit the capability of differentiation into multiple cell types, including osteoblast, adipocyte, chondrocyte, and potentially muscle cells, myocytes, neurons, and glial cells [1–6]. MSCs can be easily isolated from several adult tissues, readily expanded in vitro, and exhibit robust immunomodulatory properties. All these highly desirable attributes make MSCs a stem cell source for the development of regenerative medicines. Indeed, a huge number of preclinical studies have demonstrated promising therapeutic applications of MSCs in tissue engineering and cell-based therapy to repair and replace damaged or lost cells and tissues due to a wide variety of injury or disease, including autoimmune disorders [1–6]. The migrating or homing ability of stem cells to the desired tissues or lesions is not only crucial for normal tissue morphogenesis, homeostasis, and repair, but also for development of stem cell-based regenerative medicines [7–9].

Cell migration is a complex and highly coordinated process. Adhesive cells often migrate in the so-called mesenchymal mode, in which the migrating cell undergoes rear-to-front polarization, protrusion and adhesion formation, and rear retraction. All these major steps in cell migration are orchestrated by numerous scaffold, adaptor, and adhesion proteins (e.g., actin, myosin, integrin, paxillin, and tensin) in concerted actions that are regulated by various signaling molecules, including protein kinase C (PKC), mitogen-activated protein kinases (MAPK; c-Jun N-terminal kinase (JNK), extracellular signal-regulated kinase (ERK), and p38), Rho GTPase, Rho kinase, and focal adhesion kinase (FAK) [10–13].

Increasing MSC migration for injury or diseases might be a novel way to improve the efficiency of MSC engraftment in clinical applications. The term ‘electromagnetic fields’ (EMF) indicates a combination of electric and magnetic fields that are able to give rise to each other under certain conditions. From their time of discovery, EMF have attracted the attention of scientists as a potential therapeutic and diagnostic modality, in particular related to the application of nonionizing EMF for induction of various biological effects on cells. It has already been shown that EMF can cause changes in cell proliferation, differentiation, cell cycle, apoptosis, DNA replication and expression, cytokine expression, and more [14–22]. However, whether EMF can promote MSC migration has not yet been reported.

In the present study, we demonstrated that 50 Hz/1 mT EMF promoted MSC migration. We also found an intracellular calcium (Ca^2+^) increase following the EMF exposure, which activated FAK/Rho GTPase migratory signaling. We hypothesize that EMF treatment is an effective approach to promote the migration of MSCs to the site of injury or lesions and this has broad application prospects for regenerative medicine.

## Methods

### EMF device

**Fig. 1:**
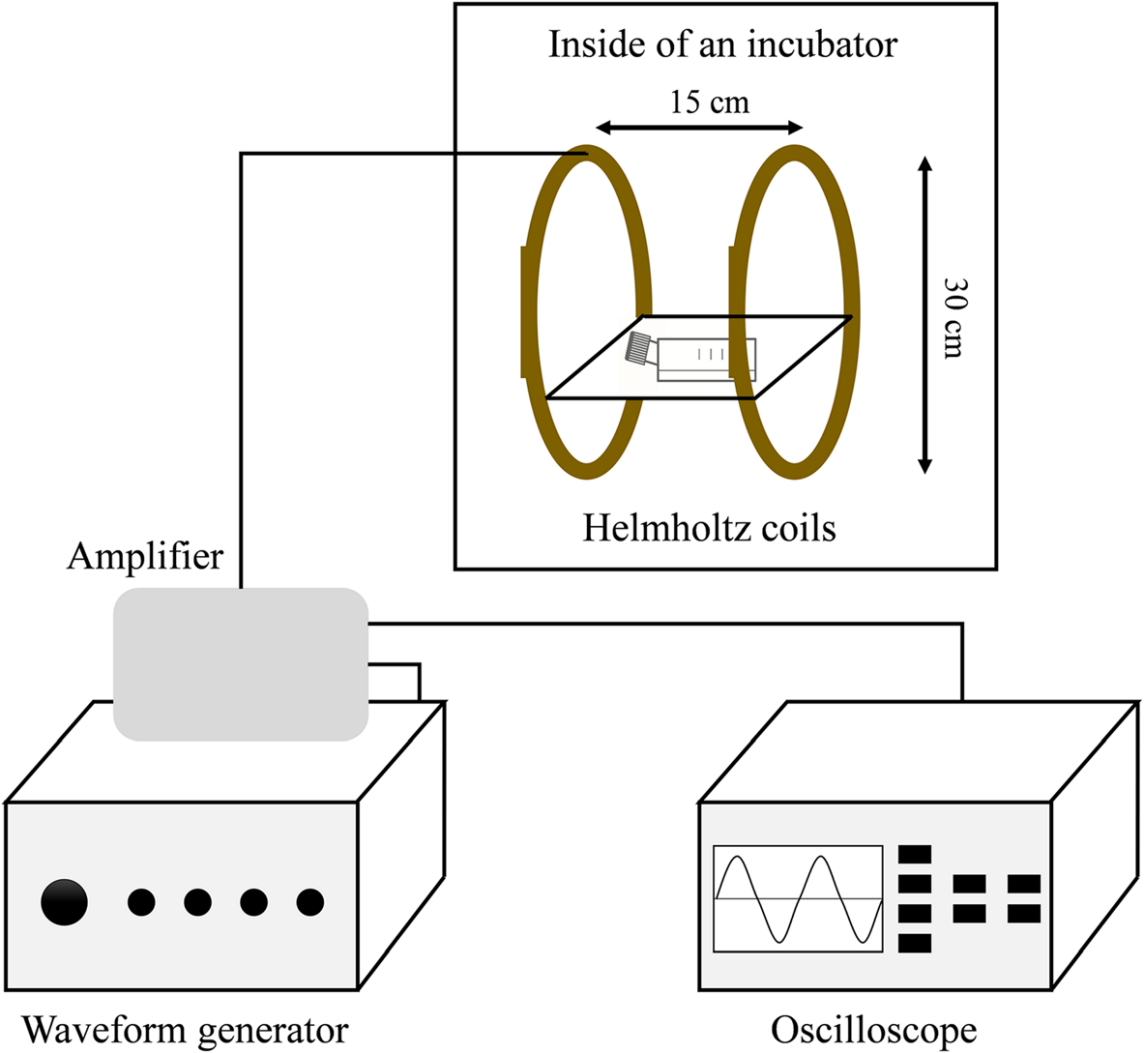
Representation of the device used to generate the electromagnetic fields (EMF). The device consists of four main parts: a waveform generator, amplifier, oscilloscope, and Helmholtz coils. The Helmholtz coils were placed in a 5% CO_2_ and 37 °C incubator. Cells were placed in the center of the coils where the EMF is uniform (90%) and the temperature is nearly the same as other incubators (within 0.2–0.8 °C)

The EMF-producing device was designed and manufactured by the Naval University of Engineering of China (Wuhan, China). It comprises a waveform generator, amplifier, oscilloscope, and Helmholtz coils. The waveform generator creates the signals which, after being amplified, are output to the coils. The Helmholtz coils producing the EMF are wound with 0.8-mm diameter coated copper wire. The coils are 30 cm in diameter, 15 cm apart, and are placed perpendicular to the horizontal plane in a CO_2_ incubator (Thermo Scientific, Wilmington, DE; 5% CO_2_, 37 °C, and 100% humidity) (Fig. 1). The device can produce a magnetic flux density range of 0.0–5.0 mT and a frequency range of 0–100 Hz. A sinusoidal electromagnetic field was introduced as this showed a satisfying effect in our previous study [23]. The magnetic field amplitude of the activated coils was measured using a gauss meter (GM55A; TinDun Industry, Shanghai, China). The uniformity of EMF was approximately 90% in the 7 cm of the spherical region (from the coil center to the origin).

### Human MSC culture and stimulation

Human bone marrow-derived MSCs (BM-MSCs) were purchased from the Cell Bank of Chinese Academy of Sciences (Shanghai, China). The cells were identified by detecting cell surface markers and the MSC multipotent potential for differentiation toward the adipogenic, osteogenic, and chondrogenic lineages (Additional file 1: Figure S1). Cells were passaged every 3 days using 0.25% trypsin (Gibco, USA) when they reached approximately 90% confluence and were used for the experimental protocols between passages 3 and 5. For the EMF treatment, MSCs were serum starved for 6–8 h, and EMF exposure was applied for 24 h. To inhibit the l-type calcium channels or activities of FAK, cells were treated with 10 μg/ml of the l-type calcium channel blocker verapamil (Sigma-Aldrich, St. Louis, MO) or 5 μg/ml FAK inhibitor (PF-573228; Sigma-Aldrich, St. Louis, MO).

### Transwell migration assay

Modified Boyden chamber assays were conducted using 24-well Transwell polyester membrane filter inserts with 8-μm pores and 0.33 cm^2^ surface area (Corning Inc., Corning, NY, USA) at a density of 500,000 cells/ml per transwell (upper chamber). The l-type calcium channel blocker verapamil (10 μM) and/or PF-573228 were used in the bottom chambers of the transwells. After culturing for 24 h, the cells from the upper chambers were removed, and the migrated cells on the undersides of the membranes were stained with crystal violet (Beyotime, Haimen, China). Migratory cells were imaged and counted in high power microscope micro-photographs (field area: 0.98 mm^2^) taken under bright light (Olympus Tokyo, Japan) using Image Pro Plus 6.0 software (Rockville, MD). 

### Cell proliferation

The proliferation of human BM-MSCs was analyzed by 3-(4,5- dimethylthiazol-2-yl)-2,5-diphenyltetrazolium (MTT; Sigma, St. Louis, MO) assay. Human BM-MSCs (2 × 10^3^) in 200 μl Dulbecco’s modified Eagle’s medium (DMEM)/F12 supplemented with 10% fetal bovine serum (FBS) were plated in 96-well culture plates. They were grown overnight and then the medium was replaced with serum-free DMEM/F12 for an additional 6 h. The cells were then treated at different frequencies of EMF (7.5, 15, 30, 50, and 75 Hz) for another 24 h. MTT (20 μl, 5 mg/ml) was added to the cells, and the plates were incubated in the CO_2_ incubator for 4 h. The resulting formazan was then solubilized in 150 μl dimethyl sulfoxide (DMSO; Sigma) and quantified by measuring the absorbance value (optical density (OD)) of each well at 565 nm. There were six duplicate wells in each group, and the experiment was repeated at least three times.

### Intracellular Ca^2+^ measurements

Human BM-MSCs grown on 20-mm diameter coverslips were loaded with Fura-2 AM in the presence of Pluronic F-127 (Sigma-Aldrich, USA) for 45 min at 37 °C. The time course of the cytosolic calcium concentration was performed at room temperature in a standard solution containing 150 mM NaCl, 5 mM KCl, 2 mM MgCl_2_, 10 mM glucose, 10 mM 4-(2-hydroxyethyl)-1-piperazineethanesulfonic acid (HEPES), and 2 mM CaCl_2_ at pH 7.3.

The basal intracellular free calcium concentration was estimated from Fura 2-AM fluorescence using dual-wavelength excitation (340 and 380 nm) and by acquiring a single emission (510 nm). The fluorescence ratio calculation and calibration were performed as reported elsewhere [24].

### Western blot analysis

The total protein from cells was extracted using RIPA lysis buffer with 1 mmol/L phenylmethylsulfonyl fluoride (PMSF; Beyotime, Haimen, China). The protein content of the cell lysates was determined using the BCA method, and approximately 20 μg of total protein was used for each sample. Protein samples were resolved by SDS-PAGE and electrophoretically transferred onto PVDF membranes (Millipore, Bedford, MA, USA). After being blocked in 5% milk or bovine serum albumin (BSA) for 2 h, the membranes were incubated with FAK, talin, vinculin, and GAPDH primary antibodies (Cell Signaling Technology, Danvers, MA, USA) at 4 °C overnight. The primary antibodies were detected with their corresponding horseradish peroxidase-conjugated secondary antibodies (Boster Biotechnology, Wuhan, China). Immunoreactive bands were obtained using a chemiluminescence imaging system (ChemiQ 4800 mini; Ouxiang, Shanghai, China). Protein levels were determined by normalizing to GAPDH.

### Rho GTPase activity assay

GTP-bound RhoA, Rac1, and Cdc42 were measured using corresponding G-LISA Activation Assay Kits (Cytoskeleton). After stimulation, cells were washed twice with cold phosphate-buffered saline (PBS) and lysed using the lysis buffer provided with the kits for 15 min on ice. The lysates were centrifuged at 10,000 × g for 1 min at 4 °C. Supernatants were aliquoted, snap-frozen in liquid nitrogen, and stored at −80 °C, as indicated by the manufacturer’s protocol. Protein concentrations were determined, and Rho GTPase activity was assessed according to the manufacturer’s instructions.

### F-actin staining by fluorescence microscopy

Cells were grown on glass coverslips until they were approximately 50% confluent and washed with PBS at 37 °C, followed by fixation with 4% paraformaldehyde in PBS for 10 min at room temperature. Cells were then washed and permeabilized with 0.5% Triton-X in PBS for 5 min. After washing, phalloidin-conjugated rhodamine (Beyotime, Haimen, China) was added at 100 nM in 200 μl PBS. After a 30-min incubation in the dark, slides were washed and stained with 4′,6-diamidino-2-phenylindole (DAPI; Beyotime, Haimen, China). After mounting with antifade mounting media, samples were examined with a microscope (Olympus) equipped with fluorescent illumination and a digital charge-coupled-device (CCD) camera. All micro-photographs were obtained under the same microscope settings.

### Statistical analysis

All values are expressed as mean values ± standard deviation. Data obtained at each time point were analyzed by one-way analysis of variance (ANOVA). Once a significant difference was detected, Bonferroni’s post-hoc analysis was used to determine the significance between every two groups. Statistical analysis was performed using the Statistical Package for Social Sciences (SPSS 15.0 for Windows; SPSS, Chicago, IL). A significance level of 95% with a *P* value of 0.05 was used in all statistical tests performed.

## Results

### EMF promoted human MSC migration

**Fig. 2:**
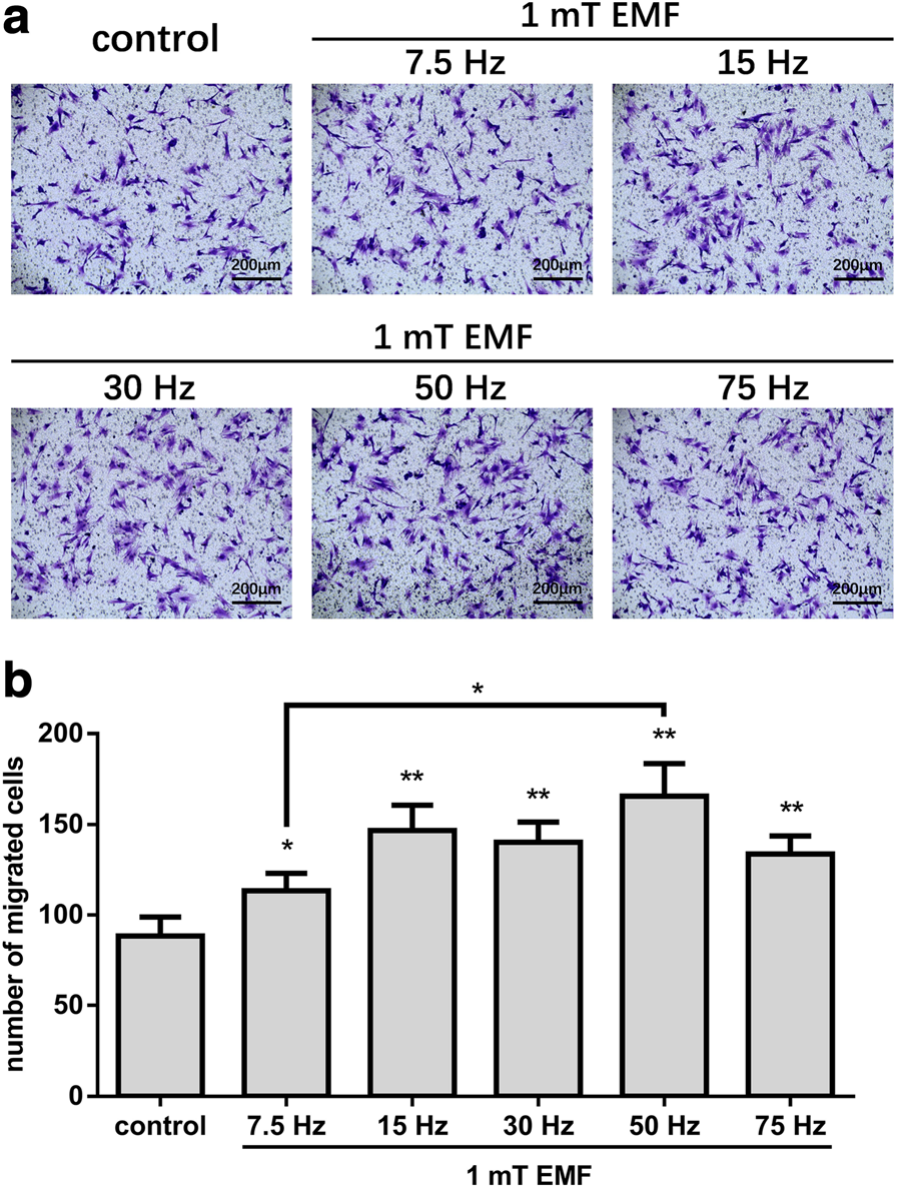
The effect of different frequencies of electromagnetic fields (EMF) on migration of human bone marrow-derived MSCs. **a** The migration ability of human BM-MSCs after stimulations with 7.5, 15, 30, 50, and 75 Hz/1 mT EMF examined using the transwell migration assay. Migrated cells on the bottom surfaces of the transwell inserts were stained with crystal violet and observed under a microscope (100×). **b** Quantitative results of cell migration. Data are presented as means ± SD. Statistically significant differences are indicated; *n* = 3; *p < 0.05, **p < 0.01, vs control or as indicated

MSCs migrating to the site of injury or lesions are an important part of tissue repair [7–9]. To explore the effect of EMF on MSC migration, we used a transwell migration assay to assess the migration of MSCs under several commonly used frequencies of 1 mT EMF exposure. We set the exposure time as 24 h according to the pre-experiments (Additional file 1: Figure S2). The results showed that EMF at all selected frequencies (7.5, 15, 30, 50, and 75 Hz) promoted MSC migration to varying degrees (Fig. 2). The 7.5-Hz EMF increased MSC migration by 26%, while the 15-, 30-, and 75-Hz EMF all increased the MSC migration by a similar amount to each other of approximately 60%. Of all the alternative EMF frequencies, 50 Hz had the most significant effect on promoting MSC migration, with an increased MSC migration of 87% (Fig. 2). The difference between the 50-Hz and 7.5-Hz groups was significant. Although there was no significant difference between the 15-Hz, 30-Hz, 50-Hz, and 75-Hz groups, the average migrated cell number in the 50-Hz group was the highest of all the treated groups (Fig. 2b). Therefore, 50 Hz/1 mT EMF was used for further research.

### EMF-promoted MSC migration is not mediated through cell proliferation

**Fig. 3:**
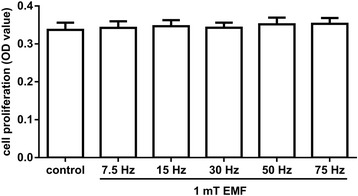
The effect of electromagnetic fields (EMF) on the proliferation of MSCs. MSCs were stimulated with different frequencies of EMF (7.5, 15, 30, 50, and 75 Hz/1 mT) for 24 h. Cells cultured under normal conditions served as the baseline. The proliferation rate of MSCs following stimulation was evaluated using the MTT assay. Data are presented as means ± SD. *n* = 3. OD, optical density

To verify whether EMF-promoted MSC migration resulted from the proliferative effects of EMF, we performed MTT assays to measure MSC proliferation after stimulations with the commonly used frequencies (7.5, 15, 30, 50, and 75 Hz) of EMF for 24 h. The results showed that EMF at all selected frequencies had no effect on MSC proliferation (Fig. 3), which suggests that the EMF-promoted MSC migration was not mediated by proliferation.

### Increased intracellular Ca^2+^ is critical for MSC migration in response to EMF

**Fig. 4:**
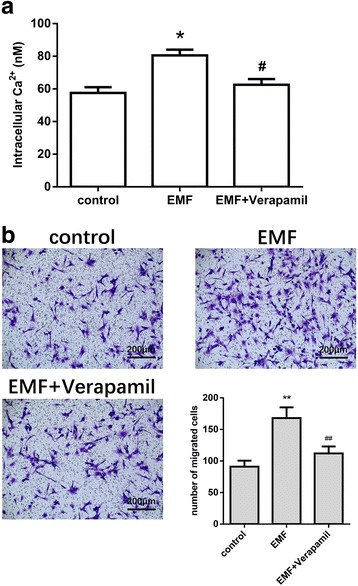
The effect of the l-type calcium channel blocker verapamil on electromagnetic field (EMF)-mediated migration of MSCs. **a** Intracellular Ca^2+^ content in MSCs after 50 Hz/1 mT EMF exposure with or without verapamil (10 μM) treatment examined using the Fura 2-AM fluorescence assay. **b** Migration ability of MSCs after 50 Hz/1 mT EMF exposure with or without verapamil (10 μM) treatment examined using the transwell migration assay. Migrated cells on the bottom surfaces of the transwell inserts were stained with crystal violet and observed under a microscope (100×). Quantitative results of cell migration. Data are presented as means ± SD. Statistically significant differences are indicated; *n* = 3; *p < 0.05, **p < 0.01, vs control; ^#^*p* < 0.05, ^##^*p* < 0.01, vs the EMF group

Cytosolic Ca^2+^ is a primary second messenger in the control and regulation of a wide range of cell functions including cell migration [25–28]. To explain why EMF promotes MSC migration, we examined the effect of EMF on intracellular Ca^2+^ content in MSCs. After 24 h of 50 Hz/1 mT EMF exposure, the intracellular Ca^2+^ increased by about 30%. Following treatment with the l-type calcium channel blocker verapamil (10 μM), EMF exposure did not significantly increase intracellular Ca^2+^ in MSCs (Fig. 4a). These results suggest that EMF may increase intracellular Ca^2+^ levels by activating l-type calcium channels.

Furthermore, we examined the effect of blocking calcium channels on MSC migration under EMF stimulation. EMF stimulation did not significantly enhance MSC migration in the presence of verapamil (Fig. 4b). These results indicate that the mechanism by which EMF promote MSC migration may be related to the increase in intracellular Ca^2+^.

### Increased intracellular Ca^2+^ after EMF exposure activated FAK and enhanced formation of focal contact in MSCs

**Fig. 5:**
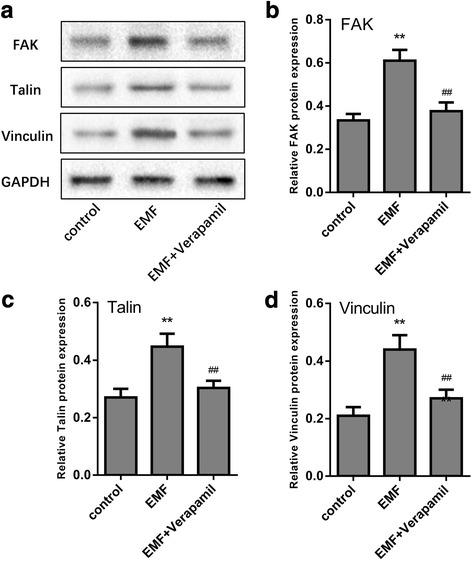
Focal adhesion kinase (FAK) contributed to the formation of focal contacts in electromagnetic field (EMF)-mediated migration of MSCs. **a**–**d** Immunoblot analysis of the total cell lysates performed using antibodies against FAK, talin, and vinculin in MSCs after 50 Hz/1 mT EMF exposure with or without verapamil (10 μM) treatment. Data are presented as means ± SD. Statistically significant differences are indicated; *n* = 3; **p < 0.01, vs control; ^##^*p* < 0.01, vs the EMF group)

Studies have suggested that FAK and its downstream proteins talin and vinculin are important for the formation of focal contacts which mediate cell adhesion [29, 30]. We therefore assessed the key adhesion proteins FAK, talin, and vinculin, by Western blot analysis after EMF stimulation following treatments with or without the calcium channel blocker verapamil. We found that 50 Hz/1 mT EMF increased the expression of FAK, talin, and vinculin, which are important proteins that form structural focal contracts. However, when stimulated with EMF in the presence of verapamil, MSCs showed significant decreases in the expression of FAK, talin, and vinculin compared with the EMF group without verapamil (Fig. 5). These data suggested that FAK was an important downstream molecule and critical for the formation of focal contacts in the EMF-increased migration of MSCs.

### Rho GTPases are activated by FAK after EMF exposure

**Fig. 6:**
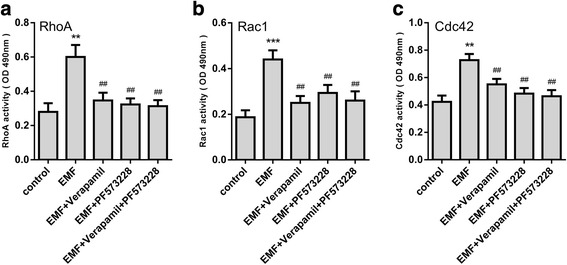
Role of intracellular Ca^2+^ and FAK on Rho GTPase activity after electromagnetic field (EMF) stimulation. A colorimetric ELISA-based assay was used to estimate **a** RhoA, **b** Rac1, and **c** Cdc42 activity levels in cell lysates. Date are presented as means ± SD. Statistically significant differences are indicated; *n* = 3; **p < 0.01, ***p < 0.001, vs control; ^##^*p* < 0.01, vs the EMF group. OD, optical density

FAK also influences the activities of the Rho-family GTPases, which participate in the dynamic remodeling of the actin cytoskeleton that drives cell migration [31–33]. To study the effect of EMF-mediated FAK on the activities of the Rho-family GTPases, the FAK inhibitor PF-573228 was used. We detected the activities of RhoA, Rac1, and Cdc42 in MSCs treated with verapamil or/and PF-573228 in the presence of EMF exposure. The results indicated that 50 Hz/1 mT EMF could significantly increase the activation of RhoA, Rac1, and Cdc42 while this change could be blocked by verapamil or PF-573228. A combined treatment with verapamil and PF-573228 did not lead to any additional inhibition (Fig. 6). These results suggested that the EMF-dependent intracellular Ca^2+^ accumulation activated the Rho-family GTPases via FAK activation.

### FAK-mediated Rho GTPase activation induces F-actin organization in MSCs after EMF exposure

**Fig. 7:**
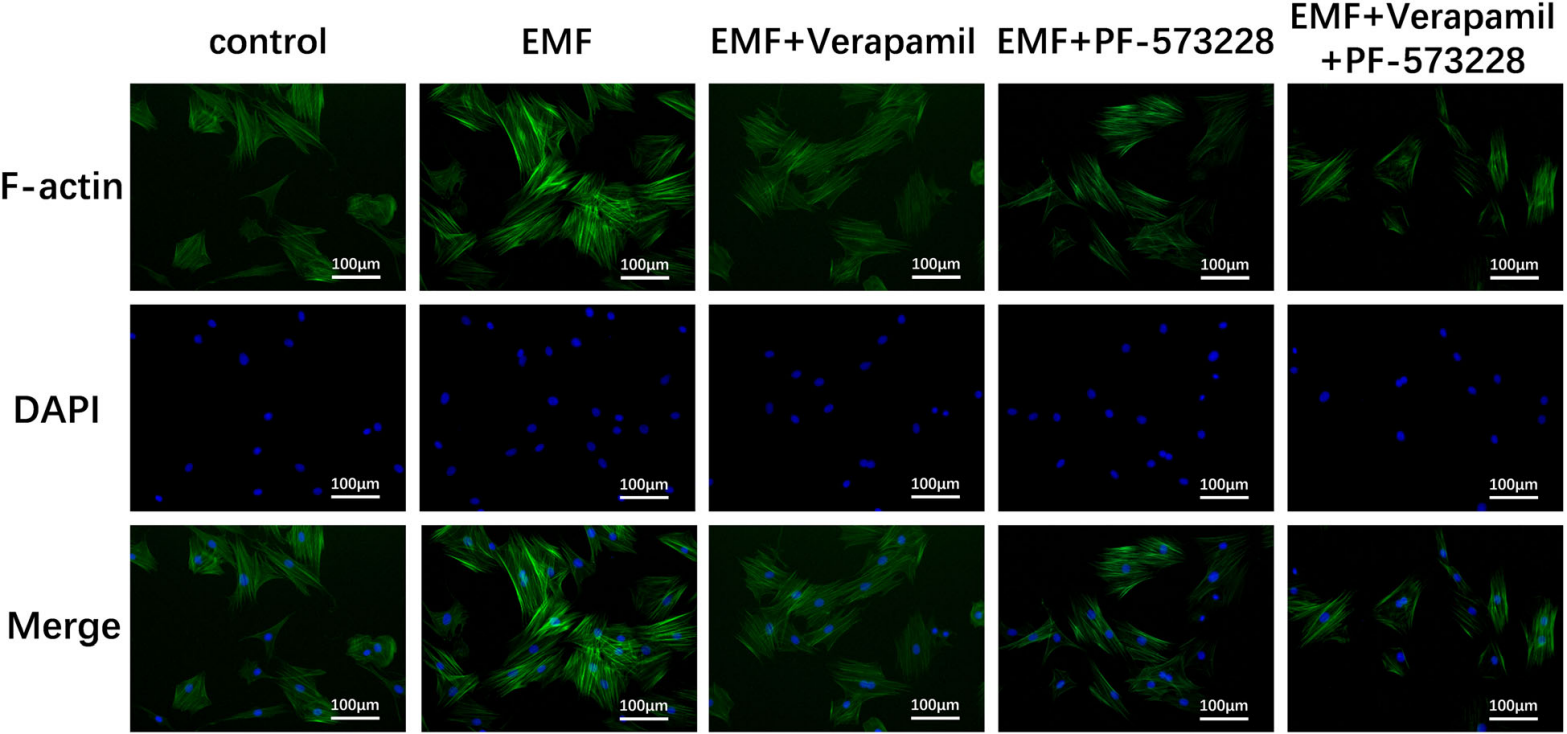
FAK-mediated Rho GTPase activation induces F-actin organization after electromagnetic field (EMF) exposure. Immunofluorescence analysis of F-actin polymerization was performed using rhodamine phalloidin (F-actin; green). Nuclei were stained with DAPI (blue). An overlay of the two fluorescent signals is shown

F-actin cytoskeleton networks, controlled by Rho GTPases, can regulate cellular shape changes and force the migration of MSCs [31, 32]. Therefore, we observed the actin structure by staining cells with rhodamine phalloidin as a probe for filamentous actin after treatments with verapamil and/or PF-573228 in presence of EMF exposure. We observed that MSCs stimulated by 50 Hz/1 mT EMF displayed more noticeable stress fibers. Meanwhile, verapamil and/or PF-573229 treatments in the presence of EMF decreased F-actin organization successfully in comparison with the single EMF group (Fig. 7). These results suggested that the EMF-dependent intracellular Ca^2+^ increase induced FAK to activate RhoA, Rac1, and Cdc42, which control F-actin organization in MSCs.

## Discussion

**Fig. 8:**
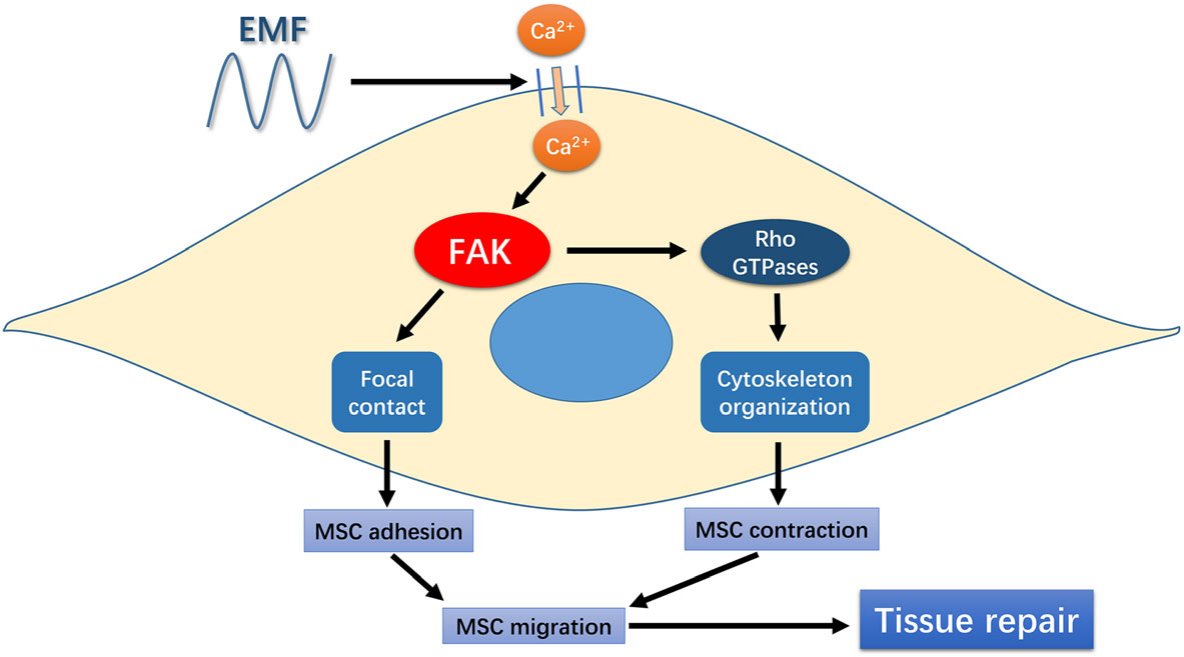
Schematic of electromagnetic field (EMF)-increased mesenchymal stem cell (MSC) migration by signaling through increased intracellular Ca^2+^ and focal adhesion kinase (FAK)/Rho GTPase pathways. EMF increases intracellular Ca^2+^. The intracellular Ca^2+^ accumulation then activates FAK, leading to the formation of focal contacts and Rho GTPase mediating the organization of the cytoskeleton, which together increases the migration of MSCs

The use of MSC transplantation to enhance therapeutic effects have been reported previously [34]. However, the invasive character of local transplantation might not be feasible for widespread clinical application. Thus, a successful systemic transplantation and the migratory ability of MSCs toward sites of injury are essential to enhance the healing process [35]. Finding a new treatment to promote MSC migration can bring new ideas to regenerative medicine and tissue engineering. EMF therapy has been proven to be an effective noninvasive approach in treating a wide range of bone diseases, such as fresh and nonunion fractures [36, 37], osteoarthritis [38], and osteoporosis [39–41], both experimentally and clinically over the past decades. Our previous studies confirmed that EMF could significantly enhance the osteoblast differentiation of MSCs [23, 42–44]. However, whether EMF can promote the migratory ability of MSCs has not yet been reported. In our study, EMF significantly enhanced the migration of MSCs (Fig. 8). In addition, the EMF-promoted MSC migration is not mediated through cell proliferation. Therefore, EMF may be an effective adjunctive treatment in regenerative medicine. However, the pro-migration effect of EMF on the MSCs was detected in the absence of any inflammatory factors released at the injury sites. Interestingly, the extent of MSC migration increased when the EMF frequency was increased from 7.5 Hz to 50 Hz, but the effect was weaker when the pulsed electromagnetic field frequency was increased from 50 Hz to 75 Hz. Our observations mimic results of Luo et al. study, which reported that 50Hz was the most effective frequency in regulating the action of MSCs [45]. However, the mechanisms associated with those variations remain unknown.

The potential mechanism of MSC migration facilitated by EMF is worthy of being investigated for EMF application. Several studies have revealed that EMF induced an increased intracellular Ca^2+^ concentration through activating voltage-gated calcium channels (VGCCs) [46, 47]. The l-type calcium channel blocker verapamil can lower or block changes in response to EMF [46, 48]. As the ubiquitous second messenger, cytosolic Ca^2+^ plays an important role in regulating many cell functions, including cell migration, and is cyto-responsive to diverse physical, chemical, and biological clues from the surrounding environment [25–28]. This motivated our investigation as to whether EMF promotes MSC migration through increasing intracellular Ca^2+^ and initiating specific downstream signaling. Our results demonstrated that EMF increased MSC migration as well as intracellular Ca^2+^, while inhibition of the EMF-dependent intracellular Ca^2+^ increase by the calcium channel blocker verapamil significantly weakened the pro-migration effect of EMF. These results indicated that increased intracellular Ca^2+^ plays an important role in EMF-stimulated MSC migration.

Cell migration is a complex and highly coordinated process. Adhesive cells often migrate in the so-called mesenchymal mode, in which the migrating cells undergo rear-to-front polarization, protrusion and adhesion formation, and rear retraction. All these major steps in cell migration are orchestrated by numerous scaffold, adaptor, and adhesion proteins (e.g., actin, myosin, integrin, paxillin, and tensin) in concerted actions that are regulated by various signaling molecules, including adhesion FAK, Rho GTPase, and Rho kinase [10–13]. FAK and the other regulators of adhesion turnover at the front appear to work at the rear as well. In addition, intracellular Ca^2+^ levels are implicated in the disassembly of adhesions at the rear. Potential targets for intracellular Ca^2+^ are the calcium-regulated phosphatase calcineurin and the calcium-activated protease calpain, which has the potential to cleave several focal adhesion proteins, including FAK, talin, and vinculin [49, 50]. We further investigated potential pathways downstream of increased intracellular Ca^2+^ that were responsible for EMF-increased MSC migration. FAK functions as an adaptor protein to recruit other focal contact proteins or their regulators, which affects the assembly or disassembly of focal contacts. Talin and vinculin, in the regulation of FAK, are important proteins that form structural focal contacts [31–33]. In this study, we found that EMF enhanced the expression of FAK, talin, and vinculin in human BM-MSCs. Moreover, blocking the calcium channel with verapamil effectively reduced EMF-induced talin and vinculin FAK expression to baseline levels, thus indicating that FAK was activated by increased intracellular Ca^2+^ in EMF-induced MSC migration.

FAK not only affects the assembly or disassembly of focal contacts but also influences the activity of Rho-family GTPases. The regulation of the Rho family of small GTPases, which includes RhoA, Rac1, and Cdc42, is essential for controlling the dynamics of the actin cytoskeleton and actin-associated adhesions during polarized cell migration [31, 32]. Therefore, with the use of the FAK inhibitor PF-573228, another important finding in our study showed that EMF not only enhanced the expression of focal adhesion proteins which facilitate cell adhesion, but also increased the activity of RhoA, Rac1, and Cdc42, which subsequently increased the formation of the F-actin network. Additionally, these changes could be reversed by calcium channel or FAK blockade. These results suggested that Rho GTPases and F-actin formation were downstream of FAK signaling in response to the EMF-induced intracellular Ca^2+^ increase.

## Conclusions

The present study firstly demonstrated that EMF increased intracellular Ca^2+^. Afterwards, the intracellular Ca^2+^ accumulation activated FAK, leading to the formation of focal contacts and Rho GTPases mediating organization of the cytoskeleton, which synergistically contributed to the increased migration of MSCs. More work is required to build an injury model and verify whether EMF can promote MSC migration to injury sites. These findings confirmed our hypothesis that EMF were capable of promoting MSC migration, which might be a promising approach to improve the therapeutic effect for many diseases.

## Additional file

Additional file 1: Human MSC culture and stimulation. (DOCX 4878 kb)

## Abbreviation

EMF: Electromagnetic fields
FAK: Focal adhesion kinase
MSC: Mesenchymal stem cell
