# Supplementary material for: Extremely low frequency electromagnetic fields promote mesenchymal stem cell migration by increasing intracellular Ca2+ and activating the FAK/Rho GTPases signaling pathways in vitro
Source: Stem Cell Res Ther. 2018 May 21;9:143. doi: 10.1186/s13287-018-0883-4 (PMC5963142; doi:10.1186/s13287-018-0883-4)
Supplement: Supplementary file 1 — Human MSC culture and stimulation. (DOCX 4878 kb) [file 13287_2018_883_MOESM1_ESM.docx]

**Identification of human bone marrow mesenchymal stem cells**

Human bone marrow MSCs were purchased from Cell Bank of Chinese Academy of Sciences (Shanghai, China). The cells were identified by detecting cell surface markers and the MSC multipotent potential for differentiation toward the adipogenic, osteogenic, and chondrogenic lineages.

Flow cytometry was performed to determine the surface markers expression on undifferentiated MSCs. We used CD105, CD73, CD90, CD45, CD34 and HLA-DR antibodies (Abcam, Cambridge, UK). The undifferentiated MSCs were positive for CD105, CD73 and CD90. CD45, CD34 and HLA-DR were not expressed in MSCs (Supplementary Fig. 1A).

To examine the multilineage capacity of MSC, cells were differentiated toward the adipogenic, osteogenic, and chondrogenic lineages using lineagespecific induction factors. To determine if MSCs undergo adipogenesis, cells were cultured in adipogenic medium (Cyagen Biosciences, Wuhan, China) and stained with Oil Red-O. MSCs cultured in adipogenic medium were induced toward the adipogenic lineage as early as 2 weeks’ post-induction. A significant fraction of the cells contained multiple, intracellular lipid-filled droplets that accumulated Oil Red-O (Supplementary Fig. 1B). Differentiation of MSC into osteoblasts was induced by osteogenic medium (Cyagen Biosciences, Wuhan, China). To confirm osteogenic differentiation, calcification of the extracellular matrix (ECM) was assessed in MSC using Alizarin red stain. Calcification appears as red regions within the cell monolayer. Consistent with osteogenesis, several red regions, indicative of a calcified ECM, were observed in MSCs treated for 2 weeks in osteogenic medium (Supplementary Fig. 1B). Chondrogenic differentiation was induced using chondrogenic medium (Cyagen Biosciences, Wuhan, China). To verify the capacity of chondrogenic differentiation, MSCs were cultured in chondrogenic medium and stained with Alcian Blue, which was used to access proteoglycans accumulation. Blue regions appeared in MSCs which cultured 2 weeks in chondrogenic medium (Supplementary Fig. 1B).

Above all, the results demonstrated that the cells used in our study possessed the phenotype expression of mesenchymal stem cells and had the multilineage capacity.
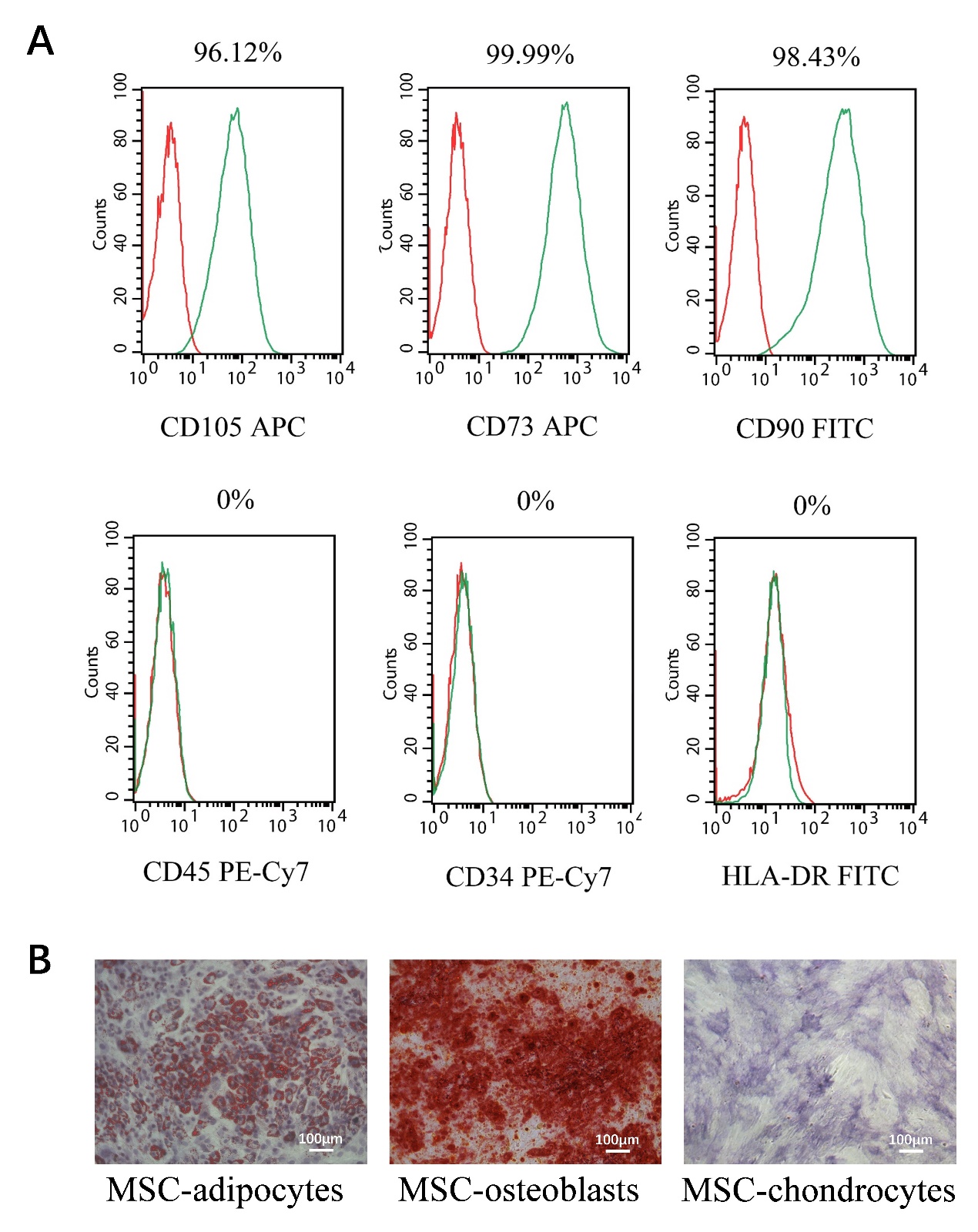


Figure S1. Identification of human bone marrow mesenchymal stem cells (MSCs) by detecting cell surface markers and multipotent potential for differentiation toward the adipogenic, osteogenic, and chondrogenic lineages. (A) Expression of mesenchymal stem cell marker was determined by flowcytometry. (B) MSCs were cultured for 2 weeks in adipogenic medium, osteogenic medium and chondogenic medium and stained with Oil Red O, Arizarin S and Alcian Blue, respectively, to identify the potential of differentiation.

**Pre-experiment to select an optimal exposure time**

To investigate the effect of electromagnetic fields (EMF) on MSC migration, we had performed a pre-experiment to select an optimal potential exposure time. Before seeding cells in the transwell plates, the MSCs were pre-treated with 50 Hz/1 mT EMF for 0 h, 1 h, 2 h, 4 h, 8 h, 16 h, 24 h, 3 d and 7 d, respectively. Our results showed that the migration levels increased time-dependently when the exposure time was raised from 0 h to 24 h. However, cell migration was not further enhanced when the exposure time increased from 24 h to 7 d (Supplementary Fig. 2). It indicated that 24 h might be an optimal exposure time for our study.


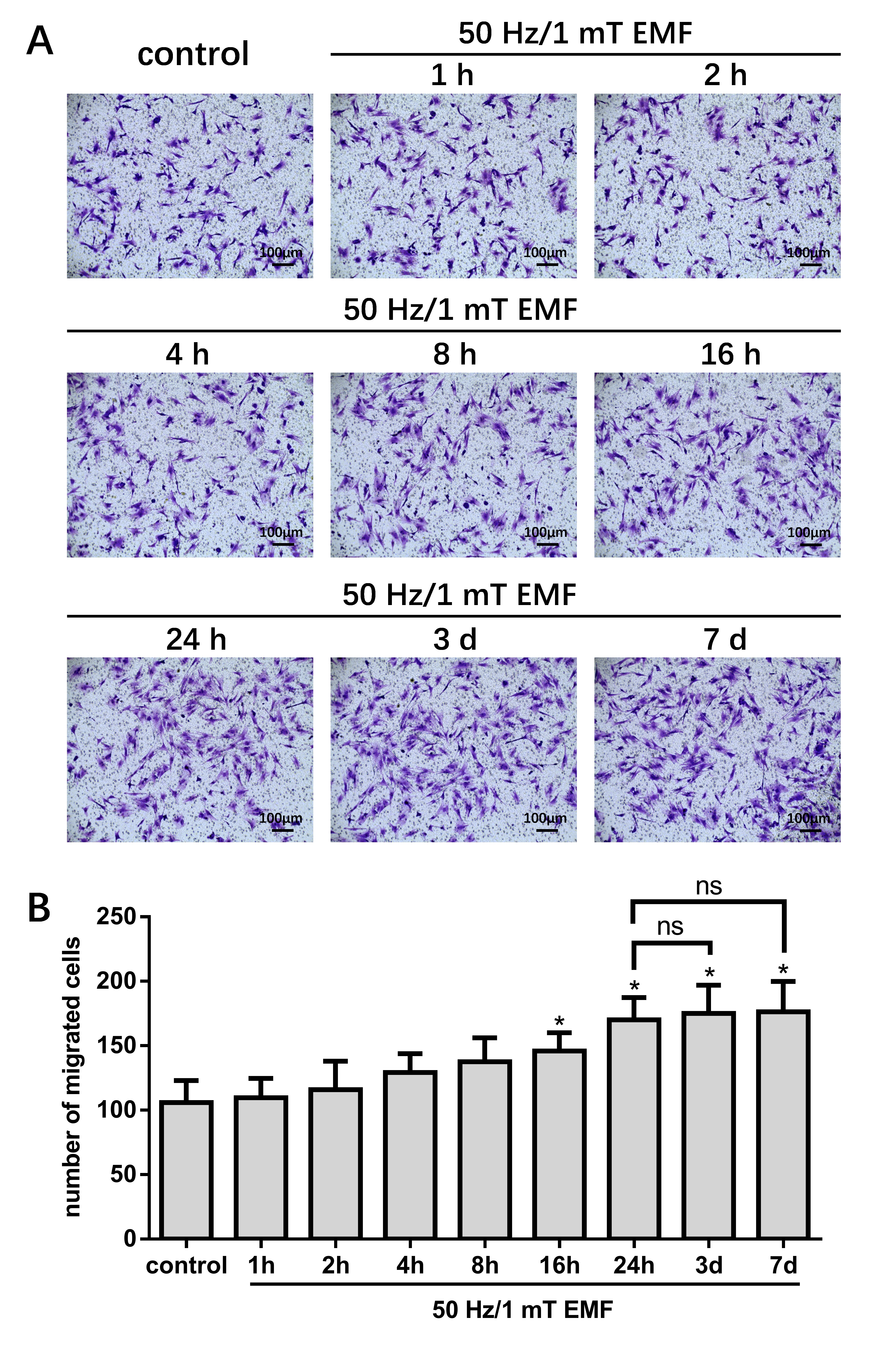


Figure S2. Effect of EMF exposure time on migration of human bone marrow cells. (A) Migration ability of human bone marrow cells pre-treated with 50 Hz/1 mT of EMF for 0 h, 1 h, 2 h, 4 h, 8 h, 16 h, 24 h, 3 d and 7 d was examined using the Transwell migration assay. Migrated cells on the bottom surfaces of the Transwell inserts were stained with crystal violet and observed under a microscope (100×). (B) Quantitative results of cell migration. n = 3; *p < 0.05, **p < 0.01, ***p < 0.001 vs control.
